# Diffusion Tensor Imaging of the Olfactory System in Older Adults With and Without Hyposmia

**DOI:** 10.3389/fnagi.2021.648598

**Published:** 2021-10-22

**Authors:** Cynthia Felix, Lana M. Chahine, James Hengenius, Honglei Chen, Andrea L. Rosso, Xiaonan Zhu, Zichun Cao, Caterina Rosano

**Affiliations:** ^1^Department of Epidemiology, University of Pittsburgh, Pittsburgh, PA, United States; ^2^Department of Neurology, University of Pittsburgh, Pittsburgh, PA, United States; ^3^Department of Epidemiology and Biostatistics, Michigan State University, East Lansing, MI, United States

**Keywords:** hyposmia, gray matter, olfaction, older adults, orbitofrontal cortex, MRI

## Abstract

**Objectives:** To compare gray matter microstructural characteristics of higher-order olfactory regions among older adults with and without hyposmia.

**Methods:** Data from the Brief Smell Identification Test (BSIT) were obtained in 1998–99 for 265 dementia-free adults from the Health, Aging, and Body Composition study (age at BSIT: 74.9 ± 2.7; 62% White; 43% male) who received 3T diffusion tensor imaging in 2006–08 [Interval of time: mean (SD): 8.01 years (0.50)], Apolipoprotein (ApoEε4) genotypes, and repeated 3MS assessments until 2011–12. Cognitive status (mild cognitive impairment, dementia, normal cognition) was adjudicated in 2011–12. Hyposmia was defined as BSIT ≤ 8. Microstructural integrity was quantified by mean diffusivity (MD) in regions of the primary olfactory cortex amygdala, orbitofrontal cortex (including olfactory cortex, gyrus rectus, the orbital parts of the superior, middle, and inferior frontal gyri, medial orbital part of the superior frontal gyrus), and hippocampus. Multivariable regression models were adjusted for total brain atrophy, demographics, cognitive status, and ApoEε4 genotype.

**Results:** Hyposmia in 1998–99 (*n* = 57, 21.59%) was significantly associated with greater MD in 2006–08, specifically in the orbital part of the middle frontal gyrus, and amygdala, on the right [adjusted beta (p value): 0.414 (*0.01*); 0.527 (*0.01*); respectively].

**Conclusion:** Older adults with higher mean diffusivity in regions important for olfaction are more likely to have hyposmia up to ten years prior. Future studies should address whether hyposmia can serve as an early biomarker of brain microstructural abnormalities for older adults with a range of cognitive functions, including those with normal cognition.

## Introduction

A reduced sense of smell or hyposmia is common in older adults, (Doty and Kamath, 2014) with more than 60% of those aged 65 to 80 years and more than 80% of those over 80 having reduced olfaction compared to younger age groups (Doty et al., 1984). A reduction in odor identification may result from abnormalities in various parts of the olfactory system, both peripheral and central, but converging evidence indicates that at least in a subset of individuals, abnormalities in olfaction result from neurodegeneration of central olfactory pathways. The central olfactory structures include the primary olfactory cortex (amygdala, anterior olfactory nucleus, piriformis cortex) and the orbitofrontal cortex (OFC) (Barresi et al., 2012).

Many of olfactory-related regions are also important for cognition and memory, and are vulnerable to neuropathological abnormalities, including amyloid accumulation, small vessel disease, and alpha-synuclein inclusions (Braak et al., 2003; Rahayel et al., 2018). For these reasons, hyposmia is considered a prodromal marker of neurodegenerative diseases, especially Alzheimer’s disease (AD) and Parkinson’s disease (PD) (Tabert et al., 2005; Baba et al., 2012; Barber et al., 2017; Chen et al., 2017; Yaffe et al., 2017). Onset of olfactory dysfunction may pre-date the diagnosis of neurodegenerative disorders by years and perhaps decades. Olfactory impairment is found to be associated with increased risk of dementia among community-dwelling older adults (Yaffe et al., 2017). In addition, olfactory dysfunction in otherwise healthy older adults (Berendse et al., 2001; Ponsen et al., 2004; Ross et al., 2008; Chen et al., 2017) and those with other prodromal features (Mahlknecht et al., 2015) predicts incident PD (Siderowf et al., 2012; Jennings et al., 2017). A post-mortem study of community-dwelling older adults found impaired odor identification to be associated with increased density of neurofibrillary tangles in the entorhinal cortex and hippocampus (Wilson et al., 2007). Initial evidence suggests olfactory abnormalities may predict brain beta-amyloid via PET (Rahayel et al., 2018).

Neuroimaging volumetric studies support the notion that olfactory dysfunction in older adults is a prodromal marker of neurodegeneration in areas that are important both for memory and olfaction. Brain volumetric studies of older adults without dementia showed worse olfactory function was associated with smaller volumes of hippocampus, entorhinal and perirhinal cortices, (Segura et al., 2013; Devanand et al., 2015; Dintica et al., 2019). Olfactory deficits in patients with early AD and mild cognitive impairment, were associated with lower hippocampal and amygdala volumes (Kjelvik et al., 2014).

While prior imaging studies have focused on AD or PD-related regions, higher-order olfactory regions, including the orbitofrontal cortex, have received less attention; one functional neuroimaging study highlighted the role of this group of regions in early aging (Wang et al., 2017). Moreover, studies have for the most part relied on volumetric measures, which capture advanced neurodegenerative stages.

Diffusion tensor imaging (DTI) is a neuroimaging sequence sensitive to microstructural and cellular changes occurring prior to volumetric changes (Alexander et al., 2007). We characterize the association between hyposmia and DTI-derived measures of olfactory regions, in community-dwelling older adults who were dementia-free at the time of olfaction testing.

## Methods

### Study Population

The study sample was obtained from participants enrolled in the community-based Health, Aging and Body Composition (Health ABC) study. The Health ABC study was a prospective cohort of Black and White men and women aged 70 to 79, on whom functional decline, especially changes in body composition with age, was studied. It started in 1997–1998 and had 3075 participants from two sites, Pittsburgh, PA and Memphis, TN (Simonsick et al., 2001).

Criteria for enrollment included: (i) no difficulty to walk a quarter of a mile, climb 10 steps, or perform activities of daily living; (ii) free of life-threatening cancers, and (iii) plan to reside in the area for 3 years (Simonsick et al., 2001). Of the 1,527 Pittsburgh site participants, 819 who returned for a follow-up visit in 2006–2008 were offered to participate in an ancillary study called the Healthy Brain Project (Rosano et al., 2015). There were 314 Health ABC study participants at the Pittsburgh site who were eligible to undergo 3T DTI MRI, and could walk 20 m (Rosano et al., 2012). Of these 314, 50 were excluded due to missing or incomplete DTI MRI mean diffusivity (MD) data (*n* = 29), missing or unreliable Brief Smell Identification Test (*n* = 10), or Modified Mini-Mental State (3MS) test score of <80 (*n* = 10) at the time of BSIT testing.

### Standard Protocol Approvals, Registrations, and Patient Consent

The institutional review boards of all the participating institutions approved the Health ABC study protocol. All participants provided informed consent (Rosano et al., 2012).

### The Brief Smell Identification Test (BSIT Sensonics, Inc., Haddon Heights, NJ)

The BSIT is a 12-item cross-cultural, rapid odor identification test, developed from the larger 40-item University of Pennsylvania Smell Identification Test (UPSIT) (Doty and Kamath, 2014). It utilizes a non-invasive method of ‘scratch ‘n sniff’ using odorant strips, with scores ranging from 0 to 12. Participants are asked to smell the strips containing an odorant, and selectively identify an odor from a list of four multiple choices. Higher scores indicate better olfaction. A score of ≤8 is deemed abnormal across ages and genders. Based on this cut-point, we identified adults as having “hyposmia” (Doty, 1995; Doty et al., 1996; El Rassi et al., 2016; Chen et al., 2017; Yaffe et al., 2017), (score ≤ 8) or no hyposmia (score > 8). BSIT was tested in 1999–2000, that is in Health ABC Year 3 (Chen et al., 2017).

### Neuroimaging

MRI scanning was performed during 2006–08 (Health ABC years 10, 11, and 12) using a 3T Siemens scanner with a 12-channel head coil, at the University of Pittsburgh’s MR Research Center. That is, MRI was performed at a mean of 8.01 years (SD: 0.50) after BSIT. Axial T1-weighted images were obtained using these parameters: TR = 2300 ms; TE = 3.43 ms; TI = 900 ms; flip angle = 9; slice thickness = 1 mm; FOV = 256^∗^224 mm; voxel size = 1 mm^∗^1 mm; matrix size = 256^∗^224; and number of slices = 176.

Diffusion-weighted imaging parameters used for DTI are as follows: single-short spin-echo sequence; TR = 5300 ms; TE = 88 ms; TI = 2500 ms; flip angle = 90; FOV = 256^∗^256 mm; two diffusion values of b = 0 and 1000 s/mm; 12 diffusion directions; four repeats; 40 slices; matrix size = 128^∗^128; voxel size = 2 mm^∗^2 mm; slice thickness = 3 mm and GRAPPA = 2.

Eddy current correction and motion correction of diffusion-weighted images were performed using FMRIB’s eddy software, which simultaneously corrects for volume-to-volume subject motion and eddy current distortions (Andersson and Sotiropoulos, 2016). Due to the lack of *b*_0_ field-maps for subjects, we were unable to perform susceptibility distortion correction on these data.

Mean diffusivity (MD) (mm^2^/s) was obtained by processing diffusion-weighted images in FMRIB’s Diffusion Toolbox (FDT) using the DTIFIT tool (Behrens et al., 2003). Partial volume effects of cerebrospinal fluid (CSF) can artificially inflate MD values in cortical voxels and cortical atrophy can increase this effect (Metzler-Baddeley et al., 2012). Free water elimination was performed using a modified version of Pasternak et al.’s method to account for cortical signal contamination by CSF (Pasternak et al., 2009). Briefly, the partial volume fraction of CSF in each diffusion-weighted image voxel was estimated from the T1-weighted structural image using FMRIB’s Automated Segmentation Tool (FAST) (Zhang et al., 2001). The isotropic “free water” component of the diffusion signal was subtracted prior to tensor fitting and MD calculation by DTIFIT (see supplement for more details).

Mean diffusivity (MD) map alignment to the MNI template was performed as follows. FMRIB’s Linear Image Registration Tool (FLIRT) was used to register the T1-weighted structural image and the diffusion-weighted *b*_0_ image (Jenkinson et al., 2002). FMRIB’s Non-linear Image Registration Tool (FNIRT) (Jenkinson et al., 2002) was then used to register the T1-weighted structural image to the T1-weighted 2mm-resolution MNI template. The resulting linear and non-linear transformations were combined to bring the MD maps into MNI space.

For this study, we chose brain regions of interest (ROIs) based on them being part of the described olfactory pathway in humans (Albers et al., 2006; Ibarretxe-Bilbao et al., 2010), and used labels based on Tzourio’s AAL parcellations (Tzourio-Mazoyer et al., 2002). Based on the neuroimaging data available, these regions included: amygdalae because of its role in primary olfactory cortex, regions of the OFC (including olfactory cortex; orbital parts of the superior, middle and inferior frontal gyri; the medial orbital part of the superior frontal gyrus; and the gyrus rectus); we also included the hippocampus because of previously shown associations.

Finally, a gray matter mask (also derived from FAST processing of the T1-weighted image) was applied to the cortical ROIs. This step was performed to minimize the contribution of white matter and CSF to MD values extracted from our ROIs.MD values were extracted from the MNI-aligned and gray matter-masked MD maps using these AAL ROIs.

MD is a reflection of cell membrane integrity and higher values may reflect underlying cell membrane damage (Alexander et al., 2007), the presence of perivascular lacunae (Sepehrband et al., 2019), or other tissue disruption (LeBihan, 2003).

### Covariates

Total gray matter volume divided by intracranial volume, indicating gray matter atrophy, 3MS and age at the time of BSIT testing (Health ABC Year 3), gender, race, education, and ApoEε4 were considered as potential confounders.

### Cognitive Status

Repeated measures of 3MS were obtained in years 1, 3, 5, 7, 9 and annually thereafter. Cognitive decline was estimated via linear mixed model with random intercepts and random slopes, using all available data from time of BSIT to the time of MRI.

In 2011–12 (Year 14 of the Health ABC study) a consensus-committee based adjudication of cognitive status was performed based on an extensive review of data collected from Health ABC study since 1998–99 including: cognitive function, mobility and disability, medical records, hospitalizations, medications, and physician’s diagnoses. All data were reviewed by a group of investigators with expertise in neurology (including AD and PD experts), psychiatry, neuroradiology, geriatric medicine, cognitive psychology, gerontology, and epidemiology. Participants were designated as having mild cognitive impairment, dementia, or being cognitively normal. Participants who had died or were in nursing homes after the MRI and by the time of adjudication were classified as a fourth group labeled as “dead/no diagnosis.” The PD adjudication was also performed separately in 2015 based on consensus review by neurologists (Chen et al., 2017). Given the small number of PD cases (*n* = 7), these participants were included in the dementia category.

### Statistical Analyses

We tested bivariate associations of hyposmia (yes/no) with population characteristics at time of BSIT, and with neuroimaging or cognitive status, using Chi-squared test or analysis of variance, for categorical and continuous variables, respectively.

The regions significantly associated with hyposmia at a significance of *p* < 0.05, were considered for inclusion in multivariable linear regression models. In the multivariable linear regression models, each brain region of interest was the outcome, hyposmia was the predictor, and each of the following covariates was entered into separate regression models: normalized gray matter volume of total brain, cognitive status (MCI, dementia, cognitively normal) age at time of BSIT testing, gender, race, education, and ApoEε4. A final model had all covariates entered simultaneously. In sensitivity analyses, 3MS at time of neuroimaging, or at time of BSIT, or decline from BSIT to neuroimaging entered the model instead of adjudicated cognitive status. Statistical analyses were conducted using SAS software version 9.4.

## Results

In this diverse sample with 43% males and 38% Blacks, about one-quarter had hyposmia (Table 1). The mean BSIT for the hyposmia group was 6.6 [SD: 1.03]. Those who had hyposmia were almost the same age as that of those who did not. Cognitive function, measured using 3MS, was significantly lower in the hyposmia group as compared to the no hyposmia group, both at the time of BSIT (*p* = *0.026*) and at the time of MRIs (*p* = *0.045*). However, the rate of cognitive decline appeared similar between the two groups (*p* = 0.354). Among the participants with cognitive adjudication in 2011–12, prevalence of dementia was 16% for those without hyposmia and 24% for those with hyposmia.

**TABLE 1:** Participant characteristics and associations with hyposmia.

		Hyposmia in 1998-1999	
		
	Analytic Sample (*n* = 265)	Yes (*n* = 57)	No (*n* = 208)
BSIT* mean, (SD)	9.8 (2.04)	6.6 (1.6)	10.6 (1.0)
Age mean, (SD)	74.9 (2.7)	75.0 (3.1)	74.8 (2.6)
Race (White), N (%)	165 (62.2)	36 (63.2)	129 (62.0)
Gender (male), N (%)	114 (43.0)	81 (38.9)	33 (57.9)
Education, N (%)			
Less than HS	31 (11.7)	8 (14.0)	23 (11.1)
HS grad	90 (34.0)	19 (33.3)	71 (34.1)
Postsecondary	144 (54.3)	30 (52.6)	114 (54.8)
ApoEε4, N (%)	66 (26.3)	15 (27.8)	51 (25.9)
Current smoker, N (%)	10 (3.8)	1 (1.8)	9 (4.3)
Hypertension, N (%)	137 (51.9)	28 (49.1)	110 (52.9)
Diabetes, N (%)	106 (40.2)	25 (43.9)	81 (39.1)
Stroke, N (%)	25 (9.4)	5 (8.8)	20 (9.6)
Head injury, N (%)	17 (6.4)	5 (8.8)	12 (5.8)
Recent cold, N (%)	22 (8.3)	6 (10.5)	16 (7.7)
3MS, mean (SD)	94.2 (4.5)	93.0 (4.9)	94.5 (4.4)
3MS, mean (SD) in 2006-2008	93.7 (5.7)	92.5 (6.5)	94.1 (5.4)
3MS, mean slope (SD) from 1998-99 to 2006-2008	–0.1 (0.3)	–0.2 (0.3)	–0.1 (0.3)
Cognitive status, 2011-2012, N (%)			
Normal Cognition	91 (34.4)	12 (21.1)	79 (38.0)
Mild Cognitive impairment	66 (24.9)	11 (19.3)	55 (26.4)
Dementia	49 (18.5)	14 (24.6)	35 (16.8)
Dead/no adjudication	59 (22.3)	20 (35.1)	39 (18.8)
Normalized gray matter volume of total brain, 2006-2008, mean (SD)	0.28 (0.02)	0.3 (0.02)	0.3 (0.02)
Interval of time between BSIT and MRI in 2006-08 (mean, SD)	8.01 (0.5)	8.01 (0.5)	8.00 (0.5)
Interval of time between BSIT and cognitive assessment in 2011-12 (mean, SD)^#^	12.66 (0.2)	12.6 (0.2)	12.7 (0.2)

*Data are concurrent with Brief Smell Identification Test (BSIT), except as described otherwise.*

*^#^ *n* = 200.*

In bivariate analysis comparing those with hyposmia compared to those without, mean gray matter MD was significantly greater in the middle frontal gyrus, orbital part and in the amygdala in the right hemisphere (Table 2). Between group differences in other regions were not statistically significant.

**TABLE 2:** Bivariate associations of hyposmia in 1998–99 with mean diffusivity of gray matter in 2006–08 in regions of interest.

		All cohort (SD)	Hyposmia (SD)		Between-group comparisons	
				
		(*n* = 265)	Yes (*n* = 57)	No (*n* = 208)	Mean difference	*p* value
**Orbitofrontal cortex (OFC):**						
Olfactory cortex	left	0.991 (0.172)	1.010 (0.954)	0.985 (0.161)	0.02	0.34
	right	0.931 (0.137)	0.919 (0.156)	0.934 (0.132)	–0.02	0.47
Superior frontal gyrus, orbital part	left	0.912 (0.141)	0.931 (0.173)	0.907 (0.131)	0.02	0.24
	right	0.936 (0.127)	0.943 (0.144)	0.934 (0.123)	0.001	0.62
Middle frontal gyrus, orbital part	left	0.830 (0.117)	0.852 (0.140)	0.824 (0.110)	0.03	0.11
	right	0.816 (0.117)	0.849 (0.141)	0.807 (0.108)	0.04	*0.02*
Inferior frontal gyrus, orbital part	left	0.943 (0.100)	0.951 (0.104)	0.940 (0.099)	0.01	0.49
	right	0.971 (0.121)	0.988 (0.141)	0.967 (0.115)	0.02	0.24
Superior frontal gyrus, medial orbital	left	0.940 (0.136)	0.952 (0.134)	0.937 (0.136)	0.02	0.45
	right	0.911 (0.110)	0.925 (0.128)	0.907 (0.104)	0.02	0.26
Gyrus rectus	left	1.081 (0.176)	1.11 (0.238)	1.07 (0.154)	0.04	0.13
	right	0.984 (0.162)	1.00 (0.209)	0.980 (0.147)	0.02	0.39
Amygdala	left	0.973 (0.113)	0.976 (0.132)	0.973 (0.107)	0.003	0.83
	right	1.007 (0.146)	1.05 (0.199)	0.996 (0.126)	0.05	*0.02*
Hippocampus	left	1.256 (0.154)	1.31 (0.173)	1.30 (0.134)	0.01	0.64
	right	1.074 (0.114)	1.31 (0.126)	1.29 (0.136)	0.02	0.35
Thalamus	left	1.044 (0.084)	1.03 (0.088)	1.05 (0.083)	–0.019	0.13
	right	0.962 (0.071)	0.956 (0.072)	0.964 (0.0.071)	0.007	0.48

*Mean Diffusivity Values in units of mm^2^/s, multiplied by 1000. *p* < 0.05 are indicated in italics.*

Associations remained statistically significant in multivariable models (Table 3). Results were similar in sensitivity analyses adjusted for 3MS values instead of cognitive status (not shown).

**TABLE 3:** Results of multivariable linear regression models showing association of hyposmia in 1998–99 with mean diffusivity of gray matter in 2006–08 in selected regions of interest.

Region name	Beta (p value), (R-square)								
	
	Unadjusted	Adjusted for:							
		Normalized gray matter volume of total brain	Age^¶^	Gender	Race	Education	Cognitive status^¶^	ApoEε4	All covariates
Right middle frontal gyrus, orbital part	0.420 (*0.02*), (0.02)	0.414 (*0.01*), (0.15)	0.412 (*0.02*), (0.03)	0.339 (0.05), (0.05)	0.418 (*0.02*), (0.03)	0.428 (*0.01*), (0.03)	0.434 (*0.01*), (0.02)	0.526 (*0.003*), (0.04)	0.441 (*0.01*), (0.17)
Right Amygdala	0.521 (*0.01*), (0.02)	0.527 (*0.01*), (0.10)	0.499 (*0.02*), (0.06)	0.479 (0.03), (0.03)	0.524 (*0.02*), (0.03)	0.536 (*0.01*), (0.04)	0.552 (*0.01*), (0.03)	0.571 (*0.01*), (0.03)	0.562 (*0.01*), (0.18)

*^¶^ : at the time of Brief Smell Identification Test (BSIT) testing (Health ABC Year 3).*

*Estimates were multiplied by 10,000. *p* < 0.05 are indicated in italics.*

## Discussion

In this study of community dwelling older adults, those with greater mean diffusivity in regions important for olfaction were more likely to have had hyposmia as early 8 years prior. The amygdala and orbitofrontal cortex are both established higher-order olfactory regions. Although participants were free from dementia at the time of the olfaction testing, cognitive function worsened during subsequent years and a large proportion developed dementia, particularly so in the hyposmia group.

Increased mean diffusivity may indicate microstructural abnormalities that can be reflective of early underlying neurodegeneration (Weston et al., 2015). Thus, our findings add support to the utility of olfactory dysfunction as a biomarker of increased risk for neurodegenerative disorders, even among community-dwelling older adults with a range of cognitive function. Consistent with prior studies in this cohort (Chen et al., 2017; Yaffe et al., 2017), that found lower BSIT scores to predict incident dementia or PD, our findings suggest that there may be structural neurodegeneration in higher-order olfactory structures related to low BSIT scores.

Our finding of an association with regions of the olfactory cortex is novel. In examining the imaging correlates of olfactory loss in older adults, most studies have focused on medial temporal regions, due to their association with memory, with a primary objective to understand hyposmia as a biomarker for AD. In contrast, we have utilized a much more comprehensive approach to study the neural correlates of hyposmia. If hyposmia is one of the earliest prodromal features of neurodegeneration, structural changes in primary, and in higher order olfactory regions such as the orbitofrontal cortex, would be expected.

We did not find an association with hippocampus. This is in apparent contrast with prior neuroimaging imaging studies of older adults with hyposmia (Segura et al., 2013; Devanand et al., 2015; Vassilaki et al., 2017; Dintica et al., 2019). However, samples in prior studies have had wider age ranges including middle-aged participants, whereas ours only included participants in their 70s and early 80s. Perhaps the smaller range of MD values in our sample could explain the lack of significant associations. Other differences with prior studies challenge direct comparisons. For instance, prior studies have had cross-sectional designs with olfaction and neuroimaging measures obtained very close in time. Secondly, unlike prior volumetric studies, we investigated mean diffusivity which may have greater sensitivity to capture earlier stages of gray matter abnormalities as compared to volumetric imaging, prior to radiologically overt volumetric reductions.

Parkinson’s disease and its prodrome provide a model from which to gain insights into olfaction as a biomarker of underlying neurodegeneration in higher order olfactory structures. Olfactory dysfunction is present in over 90% of individuals with PD (Doty et al., 1988). A few volumetric MRI studies have demonstrated olfactory area atrophy in PD compared to healthy controls, with some small studies showing a possible relationship between olfactory region volumes and olfactory function in PD (Fullard et al., 2017). In addition, studies examining ROI DTI changes in PD vs. controls underscore the relevance of olfactory tracts (Scherfler et al., 2013), anterior olfactory region (Rolheiser et al., 2011), and other regions in the primary and secondary olfactory regions (Ibarretxe-Bilbao et al., 2009; Georgiopoulos et al., 2017), including middle frontal gyrus (Karagulle Kendi et al., 2008).

Strengths of our study include having a diverse sample with 43% males and 38% Blacks. We used a hypothesis-driven approach in choosing the brain ROIs, based on them being the commonly defined central olfactory structures in humans. Availability of DTI MRI data including the small parcellated regions as described by Tzourio’s AAL helped us to study smaller GM regional microstructure within larger brain regions (Felix et al., 2020) such as the OFC. Age-related atrophy can lead to increased CSF signal contamination and overestimation of MD; free water elimination allows us to mitigate the effect of CSF contamination in this older cohort. Limitations include the fact that those found eligible to undergo MRI may have had relatively lower prevalence of serious illnesses than older adults who may not have been eligible. Available data for this study limits us from defining causality, since we only have one set of neuroimaging data and we did not have repeated measures of olfaction. Lack of field maps or susceptibility distortion correction may limit the accuracy of cortex gray matter segmentation. The study population having advanced age, and being composed of healthy survivors, may contribute to selection bias, as is common in studies of such age groups.

In summary, in community-dwelling older adults, microstructural integrity in olfaction-related regions may reflect olfactory faculties, independently of cognitive status. These findings support the utility of olfactory testing as a predictive biomarker of early neurodegeneration. Future structural neuroimaging studies of hyposmia in older adults should consider including a broader set of higher-order olfactory structures such as OFC and amygdalae, to provide a more comprehensive picture of the aging olfactory system.

## Data Availability Statement

Publicly available datasets were analyzed in this study. This data can be found here: https://healthabc.nia.nih.gov/.

## Ethics Statement

The studies involving human participants were reviewed and approved by the original Health ABC and Healthy Brain studies. We performed secondary data analysis. The patients/participants provided their written informed consent to participate in this study.

## Author Contributions

CF, LC, and CR conceptualized the study and drafted the manuscript. XZ performed the statistical analysis. All co-authors interpreted the findings and edited the manuscript for critical intellectual content. JH contributed to data analysis and manuscript revision.

## Conflict of Interest

The authors declare that the research was conducted in the absence of any commercial or financial relationships that could be construed as a potential conflict of interest.

## Publisher’s Note

All claims expressed in this article are solely those of the authors and do not necessarily represent those of their affiliated organizations, or those of the publisher, the editors and the reviewers. Any product that may be evaluated in this article, or claim that may be made by its manufacturer, is not guaranteed or endorsed by the publisher.
